# The large protein ‘L’ of *Peste-des-petits-ruminants virus* exhibits RNA triphosphatase activity, the first enzyme in mRNA capping pathway

**DOI:** 10.1007/s11262-018-1617-5

**Published:** 2018-12-03

**Authors:** Mohammad Yunus Ansari, Piyush Kumar Singh, Deepa Rajagopalan, Purnima Shanmugam, Asutosh Bellur, Melkote Subbarao Shaila

**Affiliations:** 1 Department of Microbiology and Cell Biology, Indian Institute of Science, Bangalore, 560012 India; 2 Department of Biochemistry, Indian Institute of Science, Bangalore, 560012 India; 3 Department of Anatomy and Neurobiology, Northeast Ohio Medical University, Rootstown, OH 44272 USA; 4 Division of Veterinary Biotechnology, Indian Veterinary Research Institute, Bareilly, UP 243122 India

**Keywords:** *Morbillivirus*, *Peste-des-petits-ruminants virus* L protein, PPRV, mRNA capping, RNA triphosphatase, Conventional mRNA capping

## Abstract

*Peste-des-petits-ruminants* is a highly contagious and fatal disease of goats and sheep caused by non-segmented, negative strand RNA virus belonging to the *Morbillivirus* genus—*Peste-des-petits-ruminants virus* (PPRV) which is evolutionarily closely related to *Rinderpest virus* (RPV). The large protein ‘L’ of the members of this genus is a multifunctional catalytic protein, which transcribes and replicates the viral genomic RNA as well as possesses mRNA capping, methylation and polyadenylation activities; however, the detailed mechanism of mRNA capping by PPRV L protein has not been studied. We have found earlier that the L protein of RPV has RNA triphosphatase (RTPase), guanylyltransferase (GTase) and methyltransferase activities, and unlike vesicular stomatitis virus (VSV), follows the conventional pathway of mRNA capping. In the present work, using a 5′-end labelled viral RNA as substrate, we demonstrate that PPRV L protein has RTPase activity when present in the ribonucleoprotein complex of purified virus as well as recombinant L–P complex expressed in insect cells. Further, a minimal domain in the C-terminal region (aa1640–1840) of the L protein has been expressed in *E. coli* and shown to exhibit RTPase activity. The RTPase activity of PPRV L protein is metal-dependent and functions with a divalent cation, either magnesium or manganese. In addition, RTPase associated nucleotide triphosphatase activity (NTPase) of PPRV L protein is also demonstrated. This work provides the first detailed study of RTPase activity and identifies the RTPase domain of PPRV L protein.

## Introduction

*Mononegavirales* order consists of eight families of highly divergent viruses, which possess a linear, negatively-polar, single-stranded and mostly non-segmented RNA genome. The gene organization in the viral order is conserved and the genes are transcribed from a single promoter at the 3′ terminal of genome resulting in multiple capped and polyadenylated mRNAs. The replication of the virus invariably proceeds via the synthesis of an antigenome and is performed by viral encoded polymerase complex.

mRNA capping in the *Rhabdoviridae* family of viruses is thought to proceed via a novel unconventional mRNA capping pathway due to establishment of such pathway in the *Lyssavirus* and *Vesiculovirus* genera of Rhabdoviruses [1]. The finding that the *Rinderpest virus* (RPV) L protein follows the conventional mRNA capping pathway clearly demonstrated that members of *Morbillivirus* genus in *Paramyxoviridae* family need not necessarily follow the unconventional capping pathway [2].

In the present work, we demonstrate that the RNA triphosphatase (RTPase) activity is exhibited by the ribonucleoprotein complex of *Peste-des-petits-ruminants virus* (PPRV) and a domain similar to RPV L-RTPase is located on 1640–1840 a.a. region of the PPRV L protein. The domain has been cloned, expressed and shown to have RTPase activity as well as associated nucleotide triphosphatase activity (NTPase) activity.

## Materials and methods

### Materials

Strains used for the propagation of the plasmid DNA—*E. coli* DH5α, DH10BAC, for the expression of recombinant proteins cells—*E. coli* BL21 (DE3), insect cell line Sf21 and the cloning vectors pRSET-B, pFASTBAC-HTB, pGEM-3Z and pGEM–T Easy were procured from Invitrogen, USA. The restriction enzymes *Kpn*I, *Nco*I and *Hin*dIII, Calf-Intestinal Alkaline Phosphatase, *Pfu* and *Taq* polymerases were obtained from New England Biolabs, USA. ATP and T7 RNA polymerase were purchased from Fermentas, USA. Nickel-NTA beads, DEAE-cellulose columns and plasmid midi-prep kit were procured from Qiagen, USA. PD-10 columns were purchased from GE Healthcare Life Sciences, USA. Trizol, DAB (3,3′-diaminobenzidine), Lysozyme, anti-His monoclonal antibody, plasmid miniprep kit and protease inhibitor cocktail were purchased from Sigma Chemicals, USA. Isopropyl β-d-1-thiogalactopyranoside (IPTG) was acquired from GIBCO-URL, USA. An antibody was raised in rabbit earlier against recombinant PPRV L protein domain 3 (1717–2183 a.a) expressed in *E. coli* [3]. γ-P^32^-ATP and α-P^32^-ATP were purchased from BRIT, Mumbai, India. The oligonucleotides were supplied by Sigma Chemical Co., India and were used to generate in vitro transcribed RNA substrate for RTPase assays.

### Cells and viruses

Vero cell line was obtained from National Centre for Cell Sciences (NCCS), Pune, India and maintained in Dulbecco’s modified Eagles medium supplemented with 10% foetal calf serum. *Spodoptera frugiperda* (Sf21) insect cells were obtained from NCCS, Pune, India and maintained in TC-100 medium with 10% FCS. Generation of recombinant baculovirus expressing PPRV P protein has been described earlier [4]. A recombinant baculovirus expressing L protein was generated [5] employing the full-length L gene from PPRV Tu00 virus (kind gift from late Dr. T. Barrett, Pirbright Institute, UK). PPRV Sungri/1996 virus stock was provided by Dr. R. K. Singh, Indian Veterinary Research Institute and was used to infect Vero cells to amplify the virus for isolation of RNP complex.

### Isolation of RNP complex from virus-infected cells

RNP complex of PPRV from Vero cells was isolated as described previously [5]. In brief, Vero cell monolayers (10 × 100 mm dishes, 3 × 10^6^ cells/dish) were infected with PPRV virus at a MOI of 5 and incubated for 72–84 h at 37 °C when approximately 60–70% cytopathic effect was seen. Cells were washed with ice-cold PBS, scraped off and suspended in hypotonic lysis buffer (50 mM HEPES buffer, pH 8.0, 50 mM NH_4_Cl, 7 mM KCl, 4.5 mM Magnesium acetate, 1 mM DTT and 0.1% Triton X-100). The cells were allowed to swell for 5 min on ice, and complete lysis was achieved by brief sonication. The lysate was clarified by centrifugation at 12,000×*g* for 10 min at 4 °C. The supernatant was centrifuged through 4 ml of 50% glycerol in the lysis buffer for 2 h at 32,000 rpm in a Beckman SW41 rotor at 4 °C. The pellet containing RNP complex was suspended in 50 mM HEPES buffer, pH 8.0 containing 5 mM Magnesium acetate.

### Generation of recombinant baculoviruses expressing L protein and partial purification of L–P complex

The generation of recombinant Baculovirus expressing PPRV L protein and partial purification of L–P complex was done as described previously [5]. In brief, L DNA insert was released from recombinant plasmid DNA by *Kpn*I digestion and ligated with pFASTBAC-HTB vector at *Kpn*I site. The ligated DNA was introduced into DH5α strain of *E. coli* and amplified. *E. coli* DH10BAC cells were then transformed with the recombinant DNA to get recombinant bacmids. Bacmids were tested for the presence of insert DNA of correct size using M13 forward and reverse primer set. Sf21 cells were transfected with the bacmid DNA in Grace’s media. Rescued virus was harvested 72 h after the transfection from the media. The amplified virus was used to coinfect insect cells with recombinant baculovirus expressing PPRV P protein for the partial purification of L–P complex by ultracentrifugation.

### Generation of recombinant baculovirus expressing the domain 3 (a.a. 1717–2183) of L protein

Full-length L gene from PPRV Turkey 2000 (EMBL accession no. AJ849631) was used to PCR amplify the domain 3 region corresponding to a.a. 1717–2183 on L protein. The PCR product was then used to generate recombinant bacmid and the recombinant baculovirus containing the domain 3 of L was then rescued from the bacmid in insect cells using standard procedures. The recombinant virus was then amplified in *Sf21* cells and titrated. Insect cells were infected with the virus at MOI of 5 and cells were harvested at 72 h of infection and the expression of L domain 3 was then assessed using the infected cell extracts and running SDS-PAGE and western blotting was done using domain 3 specific antibody to confirm the domain 3 expression. The antibody to domain 3 of L protein was generated using a recombinant domain 3 protein expressed in *E. coli* and purified as a His-tagged protein. This polyclonal antibody generated in rabbit reacted with full-length L protein expressed by the virus in vero cells, when tested in western blot or tested by immunoprecipitation.

### Cloning of RTPase domain

Alignment of the L protein sequence of PPRV Turkey 2000 strain with the sequences of RPV L protein and other known RTPases [2] led to the identification of a 200 a.a. (1640–1840 a.a.) long region of L protein as a putative candidate for having RTPase activity. DNA segment corresponding to the above region was PCR amplified from full-length L gene of PPRV (pGEM-3Z-L) using specific primers having sequences flanking the above region on L gene. The *Pfu* polymerase amplified PCR product was then treated with *Taq* polymerase to add template independent adenine residue at the 3′ end. The product was then cloned into pGEM–T Easy vector. The insert was subcloned into pRSET-B vector at *Nco*I and *Hin*dIII site and the resultant recombinant plasmid DNA was propagated into *E. coli* DH5α.

### Expression of the L-RTPase domain in *E. coli* and subsequent purification

*E. coli* BL21 (DE3) cells were transformed with recombinant plasmids expressing the hexahistidine-tagged RTPase domain by CaCl_2_ method and cultures were grown until the OD_600nm_ reached 0.6. The cells in the culture were incubated on ice for 20 min and induced with IPTG at 18 °C for 24 h. The cells were then harvested by centrifugation and the cell pellet was resuspended in equilibration buffer (50 mM Tris, 0.1 M NaCl, 5 mM β-mercaptoethanol, 0.01% Triton X-100 and 10% glycerol, pH 8.0) containing lysozyme (0.1 mg/ml) and protease inhibitor cocktail. The cells lysis was performed by sonication and supernatant containing the protein was collected upon centrifugation. The soluble supernatant was loaded on Ni–NTA agarose column and the resin was washed with five volumes of equilibration buffer. The protein was eluted with 150 mM Imidazole. The elution fractions containing the recombinant protein were pooled and concentrated with centrifugal filter tubes for a second-step purification with DEAE-cellulose anion exchange chromatography. The DEAE-cellulose bound protein was eluted with increasing NaCl concentrations (0.1–1 M). The fractions containing the protein were pooled, concentrated and dialyzed against buffer containing 5% glycerol, 1 mM DTT, 1 mM EDTA and 25 mM Tris (pH 8.0). Bradford concentration estimation was done and samples were stored at − 80 °C until analyzed.

### Preparation of substrate RNA

Seventeen-mer oligonucleotide (5′TAATACGACTCACTATA3′) corresponding to the T7 promoter sequence (− 17 to − 1) was annealed to the template oligonucleotide 5′CCCCGACAGTAGGATCTTTACTCCTTATAGTGAGTCGTATTA-3′ (the last 17 nucleotides are the reverse complement of T7 promoter sequence and the first 25 nucleotides are from N mRNA of PPRV). In vitro transcription was carried out using T7 RNA polymerase in standard transcription buffer (Fermentas) supplemented with 1 mM each of CTP, GTP and UTP and 0.5 µM of ATP and 10 µCi of γ-P^32^-ATP (specific activity 3500 Ci/mmol) to generate a 25 nucleotide long RNA of PPRV N mRNA, specifically labelled at the 5′ end. The transcribed RNA was passed through PD-10 columns two times to remove unincorporated radioactive nucleotide followed by ethanol precipitation.

### RTPase assay

The RTPase activity of the L protein presents a RNP complex or as recombinant domain of L protein was assessed as described earlier [3].

### NTPase assay

Standard ATPase reaction (10 µl) contained 50 mM Tris–HCl (pH 7.5), 10 mM KCl, 5 mM MgCl_2_ or MnCl_3_, 3 mM DTT, 100 µM ATP to which 0.05 µCi γ-P^32^-ATP was added with enzyme as indicated. The mixture was incubated at 37 °C for 20 min. Reactions were terminated by the addition of 1 M formic acid. Samples were spotted onto PEI-TLC sheets and developed as mentioned under RTPase assay [3]. For some experiments, a colorimetric assay for inorganic phosphate estimation was carried out as described by Noble and Neibert [6].

## Results

### PPRV ribonucleoprotein complex possesses RNA triphosphatase activity

**Fig. 1:**
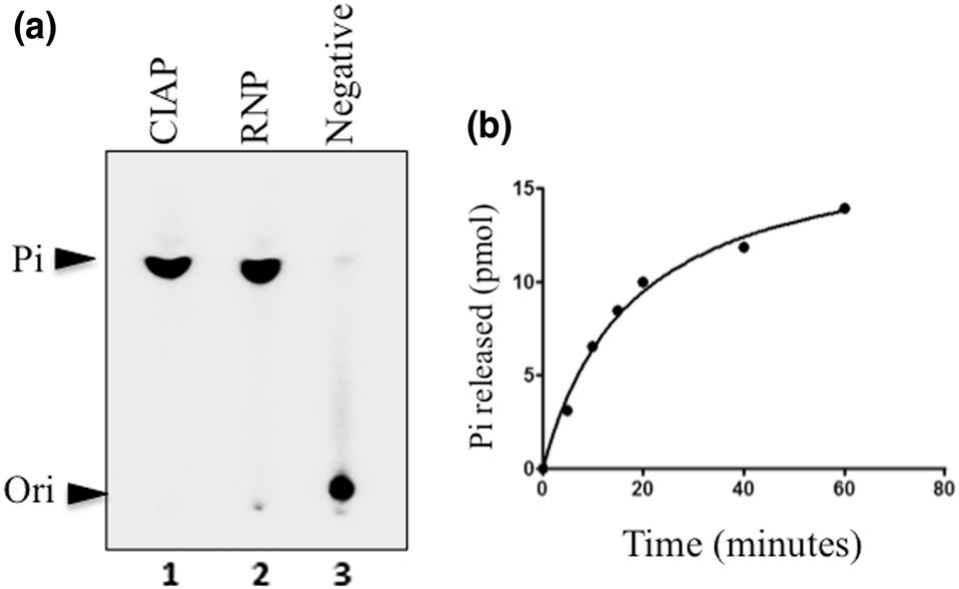
RTPase activity of RNP-associated L protein. **a** 15 pmol of γ-P32-ATP labelled RNA was incubated with 1 µg of calf-intestinal alkaline phosphatase (lane 1), purified ribonucleoprotein complex of PPRV (lane 2) and a non-specific protein (lane 3) for 30 min at 37 °C. The reaction mixtures were consequently analyzed by Thin-Layer Chromatography and Pi was detected by phosphorimaging. *Ori* origin of spotting. **b** Time course of RTPase activity. RTPase assay was performed with 15 µM of RNA as substrate and 10 µg of protein at 37 °C, pH 7.5. The reaction mixtures were incubated for various time periods and Pi released was measured by colorimetry

It has been shown that viruses of *Rhabdoviridae* family cap their mRNA employing an unconventional pathway [7] and the first enzyme in this pathway has been identified as polyribonucleotidyltransferase (PRNTase). In contrast, we had earlier demonstrated that RPV, an important member of *Paramyxoviridae* family follows conventional mRNA capping pathway and the carboxy-terminal region of the L protein of RPV carries the RTPase domain [3]. Further, this RTPase domain was expressed and its properties have been reported [2]. In order to conclusively show that the L proteins of other members in the *Morbillivirus* genus exhibit RTPase activity, we investigated if PPRV L protein has associated RTPase activity. The RNP complex was isolated from purified PPRV virus by detergent disruption and glycerol-gradient purification. The RTPase activity was assayed by incubation of γ-32P labelled triphosphate-ended RNA derived from first 25 nucleotides of N protein mRNA of PPRV with the purified RNP complex. Release of γ-32P as a result of RTPase activity was monitored by thin-layer chromatography of reaction mixture on PEI-TLC sheets. As can be seen in Fig. 1a lane 2, the Pi released due to RTPase activity co-migrated with Pi released from the same substrate when incubated with calf-intestinal alkaline phosphatase (lane 1), confirming the identity of the spot as that of inorganic phosphate. No Pi was released in the absence of RNP complex (lane 3). The extent of Pi release from 5′-end-labelled RNA substrate was proportional to the time of the reaction before saturating (Fig. 1b).

### RNP complex associated L protein possesses NTPase activity

**Fig. 2:**
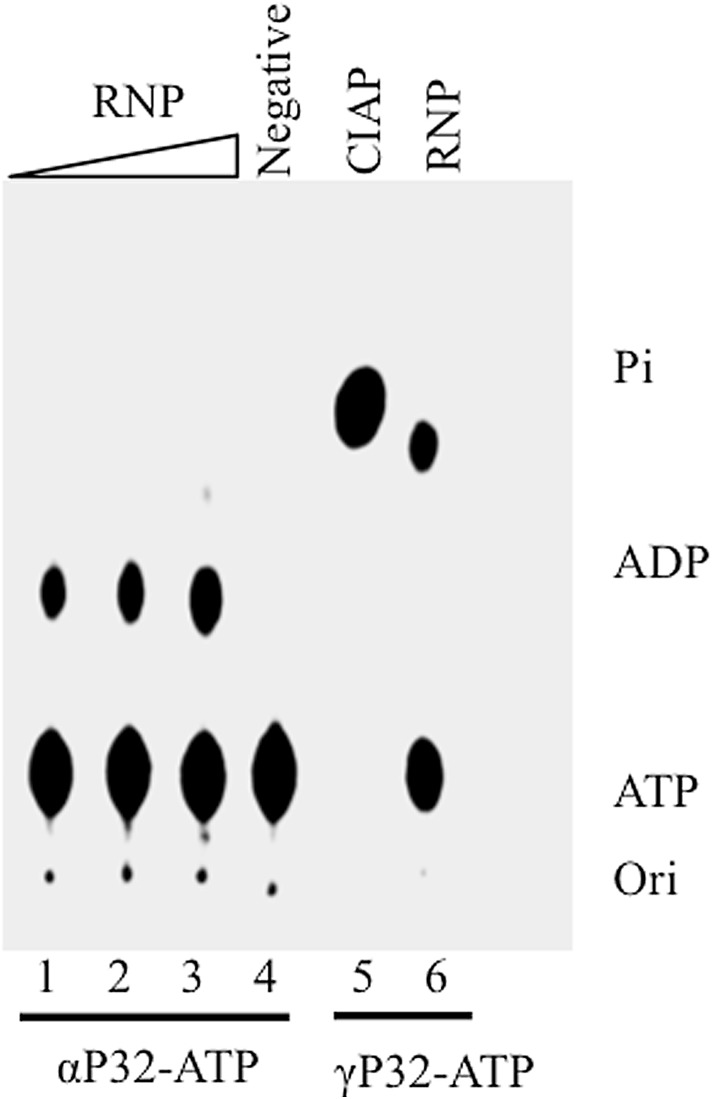
NTPase activity of RNP-associated L protein. α-P32-ATP was incubated with an increasing amount of purified ribonucleoprotein complex of PPRV (lane 1–3) with increasing yield of ADP observed on TLC. Purified fraction not showing any RNP complex in the SDS-PAGE analysis did not show any ATPase activity (lane 4). Calf-intestinal alkaline phosphatase (lane 5) and purified ribonucleoprotein complex of PPRV (lane 6) were incubated with γ-P32-ATP, both releasing radioactive Pi. *Ori* origin of spotting

Metal-dependent family RTPases have the ability to hydrolyse NTP to NDP and Pi [8–10]. We observed that the RNP-associated L protein of PPRV is also metal-dependent similar to the RTPase of RPV [2]. Since it was observed that addition of NTPs to reaction mixture having end-labelled RNA and RNP complex resulted in reduction of phosphate release, a competing NTPase activity was suspected (data not shown). In order to confirm NTPase activity of RNP-associated L protein, α-P32 or γ-P32-labelled ATP was incubated with RNP complex and Pi release was measured (Fig. 2). Labelled ADP was released from α-P32ATP (lanes 1, 2 and 3) as a function of RNP concentration whereas γ-P32 ATP released free radioactive Pi (lane 6) which co-migrated with Pi released upon incubation of γ-P32 ATP with CIAP (lane 5).

In order to demonstrate the RTPase activity of L protein devoid of other proteins of RNP complex, recombinant L protein of PPRV expressed in insect cells was employed. The rL protein was partially purified from insect cells complexed with recombinant P protein of PPRV since L protein alone is unstable, similar to the L protein of *Measles virus* (data not shown). RTPase assay was performed using r(L + P) complex. Table 1 summarizes the specific activity of RTPase of the rL protein in comparison with the RTPase specific activity of RNP-associated L protein. As can be seen in Table 1, the specific activity of RTPase of full-length L protein is higher than that of RTPase domain. This could be due to differences in folding of the recombinant full-length L protein compared to the folding of RTPase domain, since the expression systems are different.

**Table 1:** RNA triphosphatase specific activity of PPRV

Source of enzyme	Specific activity (nmoles/mg)
RNP from virus	18
Recombinant L as L–P complex	600
Purified recombinant RTPase domain	103

The values are given as the mean of six assays. All assays were performed in vitro

### A C-terminal subdomain (1640–1840 a.a.) of L protein possesses RNA triphosphatase activity

**Fig. 3:**
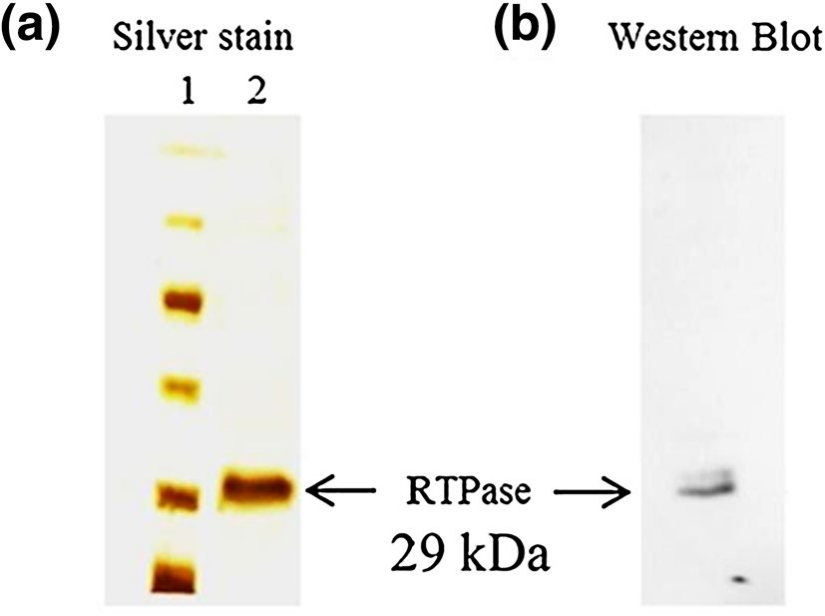
Expression of recombinant PPRV L-RTPase domain and its purification. The RTPase domain of L protein of PPRV was expressed in *E. coli* BL21 (DE3) strain and was purified with anti-His-tagged antibody affinity column. Electrophoresis was performed in 12% polyacrylamide gels. **a** Silver staining of purified RTPase. Silver staining was performed with the electrophoresed purified recombinant RTPase domain after the purification. **b** Immunoblot of purified RTPase. The electrophoresed recombinant RTPase was transferred to nitrocellulose membrane and probed with a PPRV L protein domain 3-specific polyclonal antibody raised in rabbit

**Fig. 4:**
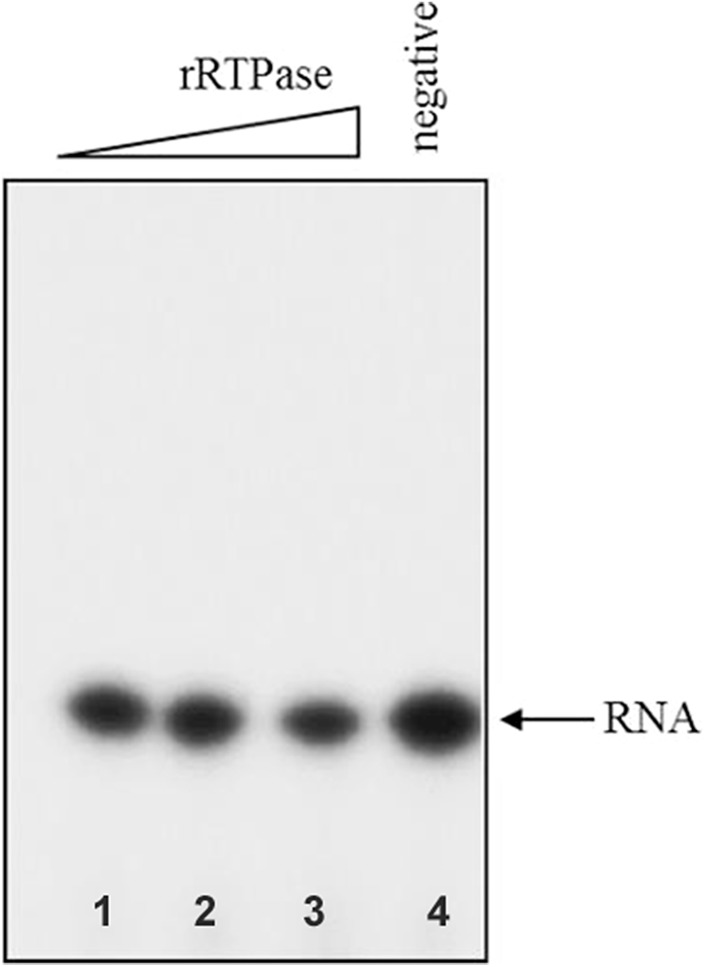
RTPase domain of the PPRV L Protein has no nuclease activity. Increasing concentrations of recombinant PPRV L-RTPase was incubated with alpha-P32 labelled RNA substrate for 30 min and was analyzed by 20% Urea-PAGE

**Fig. 5:**
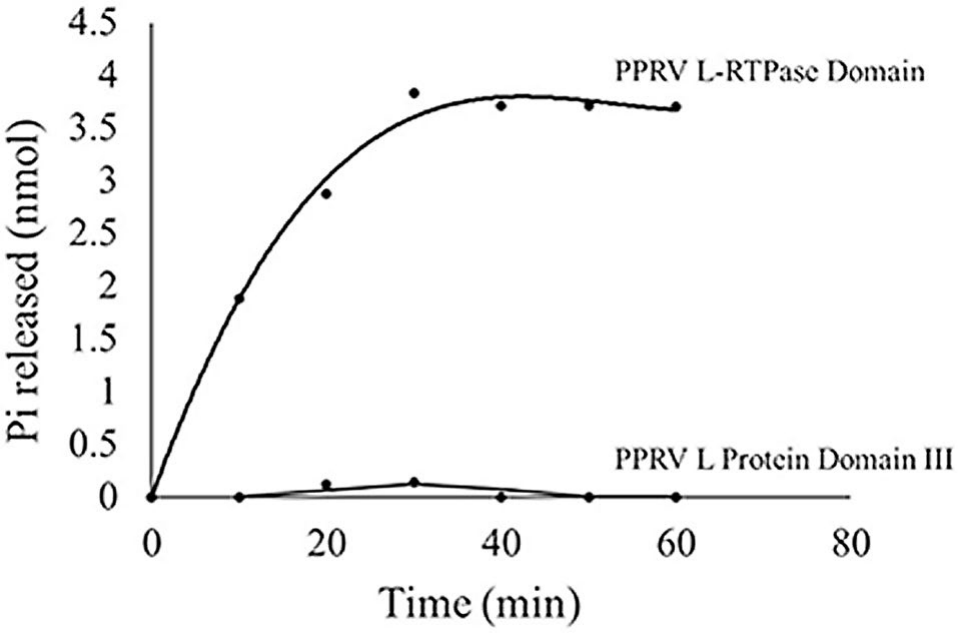
RNA triphosphatase activity of PPRV RTPase domain. γ-32P-ATP-labelled RNA was incubated with recombinant RTPase domain of PPRV L protein and with recombinant domain III of L protein for various time periods. The reaction mixture was analyzed by TLC on PEI-cellulose plates and Pi release was detected by phosphorimaging

In order to identify the domain mediating RTPase activity of L protein, multiple sequence alignment of L protein sequence with those from other members of *Morbillivirus* genus along with the known RTPases of *Vaccinia virus*, baculovirus and other viruses was performed. The characteristic feature of metal-dependent RTPase is the presence of two-metal binding pockets and a catalytic motif rich in basic amino acids. The analysis revealed a 200 amino acid long region (1640–1840 a.a.) on the C-terminal part of L protein of PPRV as a putative candidate for RTPase. To test if this region of L indeed catalyzes RTPase activity, DNA segment corresponding to the above region was PCR amplified from full-length L gene using specific primers. This amplified DNA was then cloned into an expression vector and protein expressed in *E. coli* BL21(DE3). The expressed protein was purified on an anti-His-tagged antibody affinity column. Figure 3a shows the silver-stained gel picture of the purified protein. Upon SDS polyacrylamide gel electrophoresis, the protein was immunoblotted with polyclonal antibody made earlier against recombinant L domain 3 [2]. Figure 3b shows the identity of recombinant RTPase (rRTPase) domain of molecular weight 29 kDa. The purified protein was tested for any contaminating *E. coli* derived RNase. Figure 4 shows the results of urea-polyacrylamide gel electrophoretic run of α-P32-labelled RNA substrate incubated with different amounts of purified rRTPase domain. The results clearly demonstrated that no shorter RNA fragments are seen in the gel. There was also no indication of non-specific phosphatase activity which could result in release of phosphate on TLC run of an incubated gamma-labelled substrate RNA (data not shown). The RTPase activity of rRTPase domain was then assayed with end-labelled RNA and Fig. 5 provides the data on time kinetics of RTPase activity. It was of interest to test if domain 3 of PPRV L protein in the region 1717–2183 a.a.—which also harbours the amino terminal portion of RTPase domain, is capable of showing RTPase activity. The activity profile for PPRV L protein domain 3 is shown in comparison with rRTPase domain (Fig. 5) showing that the PPRV L protein domain 3 has no RTPase activity.

**Fig. 6:**
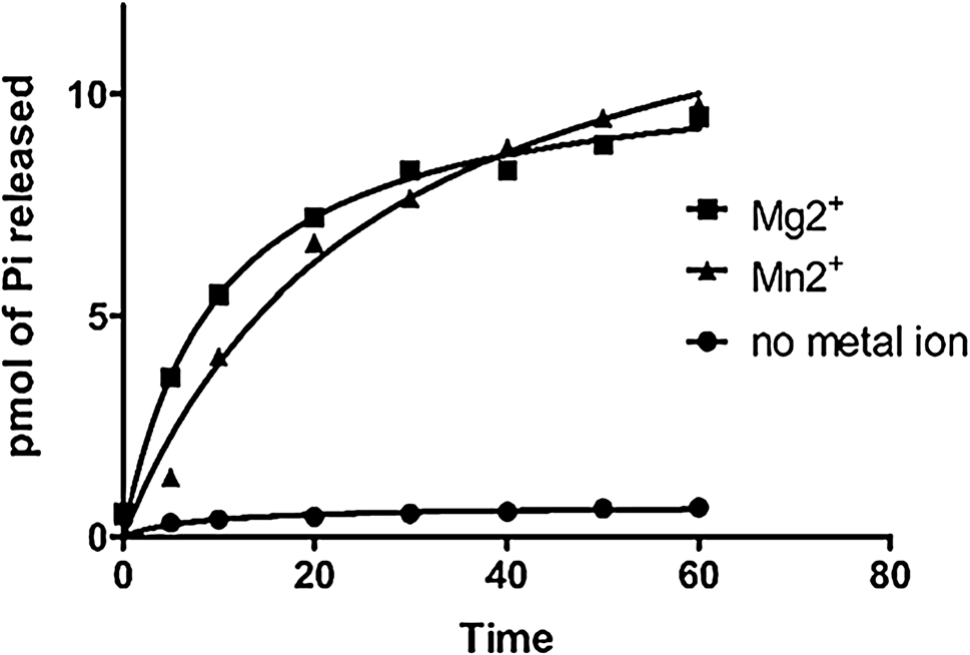
Metal dependence of the RTPase activity of recombinant PPRV L-RTPase domain. The γ-phosphate labelled RNA was incubated with recombinant L-RTPase domain in the presence of either Mg^2+^ or Mn^2+^ or no metal ion. Release of γ-phosphate was analyzed by Thin-Layer Chromatography and plotted as a function of time

The RTPase activity in the RPV is known to be dependent upon Mg or Mn divalent cation similar to the tunnel-type metal-dependent RTPase [2]. Since the PPRV is closely related to RPV and the alignment of PPRV L protein showed high sequence similarity, we investigated the dependence of PPRV rRTPase on divalent metal cations—Mg^2+^ or Mn^2+^ (Fig. 6). The enzyme is capable of functioning either with Mg^2+^ or Mn^2+^ equally well but the activity is absolutely dependent on the presence of these cations.

### Recombinant RTPase domain exhibits NTPase activity

**Fig. 7:**
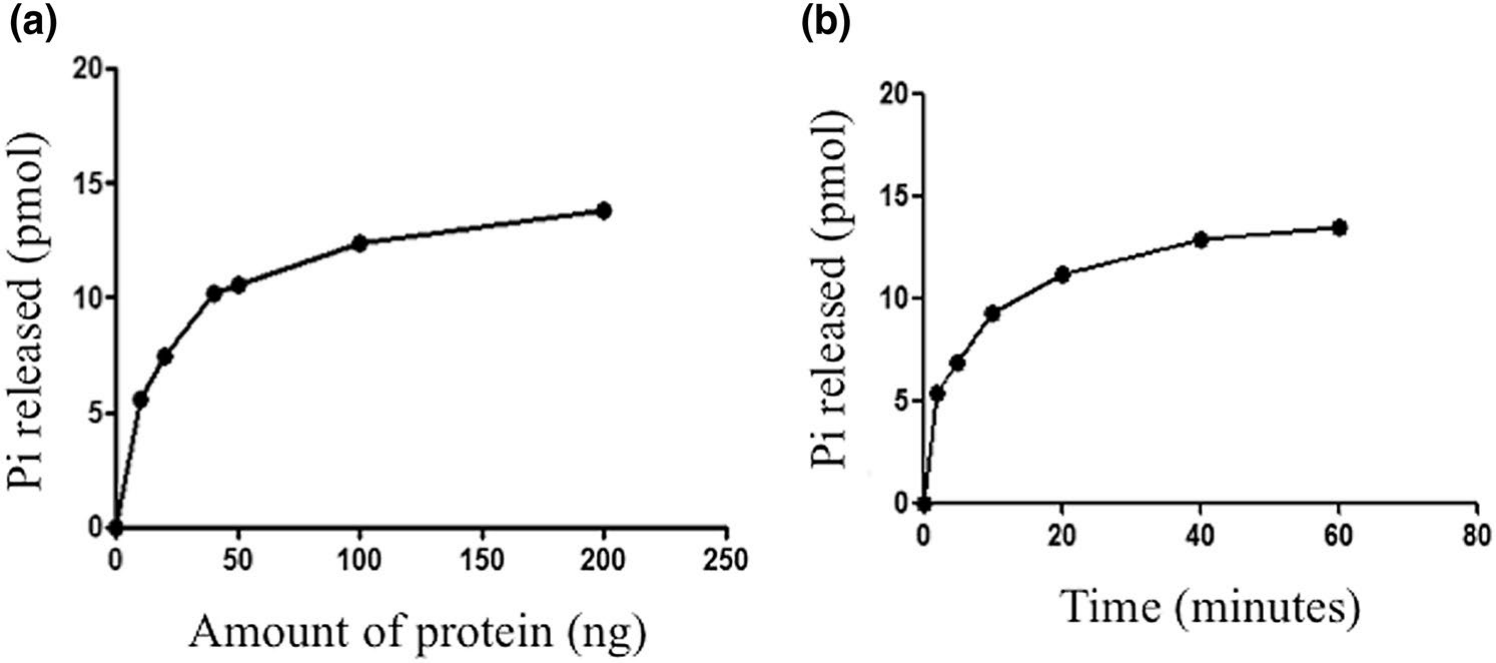
NTPase activity of the recombinant RTPase domain. **a** The ATPase assay was performed at 30 °C for 30 min with 2 mM of ATP as substrate and varying amounts of protein. **b** The time course of ATPase activity was studied with 2 mM of ATP as substrate and 5 µg of protein by performing reaction at 30 °C for various time periods

NTPase activity was tested with the rRTPase domain of PPRV. Figure 7a shows the results of NTPase activity as a function of protein concentration, while Fig. 7b shows the time kinetics of the reaction. The data show that the rRTPase domain has the NTPase activity.

Two site directed mutants (E1645A and E 1647A) of RTPase domain of L protein were generated, expressed in *E. coli* and purified. The purified mutant proteins were tested for RTPase and NTPase activities. While E1645A mutant showed 17% of wild type RTPase activity with RNA as substrate, E1647A mutant retained 30% of wild type NTPase activity, demonstrating the relative importance of E residue in the motif A for its RTPase activity. The NTPase activity of both mutants was completely lost (data not shown). These results are very similar to our earlier observed results for Rinderpest virus RTPase and NTPase activity of the RTPase domain [2].

## Discussion

The L protein of *Mononegavirales* is believed to have monophyletic origin owing to high sequence similarity, resemblance in structure and function [11]. The enzymatic activities of polymerase, mRNA capping and methylation reside in the L protein of the viruses of *Mononegavirales* order. The L protein of vesiculoviruses (*VSV* and *Chandipura virus*) as well as *Rabies virus* in the *Lyssavirus* genus, which belong to the *Rhabdoviridae* family, catalyze an unconventional mRNA capping reaction with a novel enzyme—PRNTase [7, 12, 13]. Based on the conservation of HR motif (covalent polynucleotidyl site) as well as some conserved sequence stretches in most non-segmented RNA viral L protein, it was implied that the same unconventional capping pathway exists in the viruses of *Paramyxoviridae* family [7]. However, our recent work on the capping activity of the L protein of RPV has revealed that mRNA capping is through a conventional eukaryotic pathway [2]. Our earlier work [3] had provided evidence that the L protein of RPV possesses RTPase as well as guanylyltransferase activities. In the present work, we demonstrate that the L protein of another *Morbillivirus*—PPRV also has RTPase activity located at the carboxy-terminal region of L protein, which has been expressed and activity demonstrated.

Employing comprehensive in silico and experimental approaches, Dochow et al. have demonstrated that paramyxovirus polymerases are composed of atleast two independent folding domains, L domain I (1–1708 a.a.) and L domain II (1709–2224 a.a.), which are transcomplementable [14]. It is interesting to note that the in silico predictive methods employed by the above authors failed to predict the existence of a functional subdomain occupying a position bridging both the domains. This limitation has been pointed out by the authors saying that the additional domain intersections may exist in L proteins of paramyxoviruses that were not detected in their work. Since the L domain 3 (1717–2183 a.a.) did not exhibit RTPase activity (Fig. 5), it may be surmised that for RTPase activity, the extreme carboxy-terminal region of domain 2 (as earlier identified in *Morbillivirus* L) is essential which is lacking in L domain 3. Thus, it is evident that the enzymatic function does not reside in domains defined by high sequence conservation among *Morbillivirus* L proteins or in the independently folding domains as proposed by Dochow et al. [14], Our findings on the RTPase activity of L protein of PPRV reaffirm our earlier demonstration of operation of conventional mRNA capping pathway in *Rinderpest virus*—an important member of *Morbillivirus* genus in *Paramyxoviridae* family [3], leading us to speculate that other members of this genus may also follow the conventional capping pathway. The close proximity of the functional subdomain of RTPase to the rest of extreme carboxy-terminal region having methyltransferase demonstrated with RPV L [15] is a pointer in favour of the suggestion made by Dochow et al. [14]—based on their architectural model of *Mononegavirales* L protein, that independently folding subdomains do not share traditional protein–protein interface but require low affinity molecular compatibility.

The presence of the RTPase activity in two morbilliviruses—RPV and PPRV strengthens the emerging view that the viruses of the *Paramyxoviridae* family follow conventional eukaryotic mRNA capping using virus-borne capping enzymes. Since RPV L protein has been shown to possess guanylyl transferase activity [3] it is highly likely that PPRV L protein also should be having GT activity domain, which requires to be investigated. Further functional characterization of the capping activities of other viruses of *Paramyxoviridae* family is needed before a definite conclusion can be made.
